# “Sorry, I’m not from here!”: Female international student-athletes’ transitions into college athletics in the United States

**DOI:** 10.3389/fpsyg.2023.1103194

**Published:** 2023-03-13

**Authors:** Nicholas Swim, Youngjik Lee, Mary A. Hums

**Affiliations:** ^1^Department of Health and Sport Sciences, University of Louisville, Louisville, KY, United States; ^2^Department of Physical Education, Kookmin University, Seoul, Republic of Korea

**Keywords:** international student-athletes, US college athletics, transition experiences, multiculturalism, organizational psychology

## Abstract

The number of NCAA international student-athletes (ISAs) on US college campuses has increased to upwards of 20,000. This current study sought to investigate their transition experiences into colleges, based on the ISA transition adjustment model. More specifically, this study sought to better understand how recent changes in the NCAA have impacted the ISA population and if the antecedent factors in the transition adjustment model (personal, inter-personal, perceptual, and cultural distance) still represent the best indicators of successful transition for ISAs. To conduct this study, semi-structured interviews were completed with 10 current and former female Division I ISAs from six separate schools and seven countries. The results from this study suggest the core antecedents of the model – personal, inter-personal, perceptual, and cultural distance – were all still relevant. However, the factors included among these antecedents have shifted over time, with this study finding the importance of faculty and students (inter-personal) and nutrition (cultural distance) as significant factors in the transition of ISAs into US colleges. The results provide insights to administrators of US college athletics regarding how to help international student-athletes’ adaptation.

## 1. Introduction

The number of International Student Athletes (ISAs) attending NCAA Universities has steadily increased in recent years. In the 1999–2000 academic year, across all three NCAA Divisions, just under 6,000 ISAs competed in NCAA sports (Bray, 2002). Fast-forward to the 2019–2020 academic year, and NCAA Division I and II schools featured over 22,985 ISAs from 206 different countries (National Collegiate Athletic Association, 2021), a stark exponential growth of 283%. In 2019 alone, the NCAA saw upwards of 5,500 freshman (male and female) ISAs at the Division I and II levels, demonstrating that the growth in the numbers of international students is staying consistent (National Collegiate Athletic Association, 2022). Many reasons account for the growth of ISAs on college campuses, including increased recruiting opportunities *via* social media and online settings, demand for more diverse college campuses, improved visa policies, successes of past ISAs (increased demand), and greater international exposure through national tournaments/events (Love and Kim, 2011; Cole, 2019; Hanson, 2020; Otto et al., 2020).

For years, recruiting and retaining international students have been significant initiatives for universities. This population creates diverse learning experiences, generates unique points of view, increases awareness of different cultures, and expands technical fields (STEM) which benefits the entire campus community through creating a well-rounded campus culture and atmosphere (Lee and Rice, 2007; Sherry et al., 2010; Perry, 2016). Montgomery (2010, p. 6) states, “International students are transient visitors to our academic communities, yet they form an integral part of the social, cultural, and academic context of higher education.” While some studies have found the positive experience of international students will increase as more students make the journey abroad (Beech, 2014), others suggest potentially negative experiences upon attending college as an international student (Heng, 2017, 2018; Gareis and Jalayer, 2018). As the “visitors” identification on campus may allow for differential experiences in comparison to fellow domestic students, especially during the transition process as the current US college system differs drastically from other countries’ higher education models (Hong, 2018). Spurling (2006) even suggests domestic and international students experience a tough time creating working relationships based on their perceived differences in academic exchanges (challenges with language barriers) on college campuses (Gareis and Jalayer, 2018). This lack of connection between domestic and international students may even marginalize ISAs at universities, as they remain a minority on most college campuses (Sato et al., 2011).

This marginalization of the ISA population also isolates them from non-athlete international students, as the domestic student population on college campuses accepts ISAs based primarily on their highly identifiable status as NCAA student-athletes (Foo et al., 2015; Charitodini and Kaburakis, 2022). Forbes-Mewett and Pape (2019) also argue that ISAs receive more support (academically, personally, and fiscally), as these additional support systems are often not available to other non-athlete international students (Ridinger and Pastore, 2000a,b). Even with these differentiating experiences, ISAs are more likely to transfer (25%) than domestic student-athletes (14%) and report a lesser sense of belonging than domestic student-athletes, highlighting the need to better understand the differences in experiences for ISAs (National Collegiate Athletic Association, 2021).

The first to address the varying experiences of ISAs was Ridinger and Pastore (2000a,b) who examined the transition experience into US colleges. The results of these studies developed the ISA transition adjustment model, which relied on four key antecedent factors (personal, interpersonal, perceptual, and cultural distance) that were thought to predict the overall adjustment experience for ISAs. The original adjustment model (Ridinger and Pastore, 2000a) was re-examined by Popp et al. (2010) to explore the intersection of travel and sense of adventure on the transition process. Yet, since the Popp et al. (2010) article, there has been no re-examination of the antecedents of the adjustment model. With the drastic changes in the NCAA landscape over the past 10 years (increase in number of ISAs, conference re-alignment, social media growth, NIL, etc.) a re-examination of the adjustment model is needed to better understand and explore the current ISA transition experience.

### 1.1. Review of literature

#### 1.1.1. International student-athletes

Given the increased number of ISAs since the early 2000s, many scholars have conducted research with regard to ISAs at NCAA institutions, as their experiences are vital for the continued growth of college sport internationally (Garant-Jones et al., 2008; Popp et al., 2009, 2010, 2011; Saxe et al., 2017; Baghurst et al., 2018; Otto et al., 2020; Turick et al., 2020; Hauff et al., 2021). Past research has indicated the ISA population lives unique experiences on US college campuses, as they may be subjected to any combination of struggles involving academic demands (Popp et al., 2010), athletic demands (National Collegiate Athletic Association, 2021), cultural differences (Pierce et al., 2012), homesickness (Otto et al., 2020), and language differences (Sato et al., 2011). Some ISAs even indicate they would not have attended school in the US if not for their sport participation, demonstrating the importance of athletics in their experiences (Rodriguez, 2014). This athletic focused transition likely creates stressful situations for ISAs upon their matriculation at US universities.

An ISA’s athletic focused decision to attend a US university may increase challenges to their academic endeavors (Jolly et al., 2022). One reason for this challenge can be the differences in higher education structures in the US compared to the ISA’s home country (Ridinger and Pastore, 2000a; Sato et al., 2011; Otto et al., 2020). For example, some ISAs struggle to understand terminology associated with US higher education, including course levels and year in school. Hauff et al. (2021) found the term ‘freshman’ was confusing for ISAs. Further, Otto et al. (2020) found ISAs struggled with understanding the intricacies of GPA, how/when to register for classes, and managing online education platforms. Another challenge to academic success is properly managing academic and athletic time demands due to the need for ISAs to spend extended time focusing on academic competency (Sato et al., 2011; Ridpath et al., 2020). One reason for this extended time spent on academic endeavors is the language barrier, which for non-native English-speaking ISAs, demonstrates a significant academic disadvantage (Sato et al., 2011; Otto et al., 2020).

For ISAs, another factor influencing their experiences is potential differences in culture. Upon arrival to the college, ISAs are often asked to adapt to fit into the current model/culture of college athletics, rather than allowing the system to fit them (Otto et al., 2020). These cultural differences actually become multiplied the greater the geographical distance between an individual’s home country and the US. This suggests greater adjustment struggles for ISAs from countries further away from the US (Pierce et al., 2012). For example, Canadian student-athletes competing in the US indicated easier transition experiences than those from countries a greater geographical distance away (Pierce et al., 2012). Manwell et al. (2021) illustrated that Hispanic ISAs are usually struggling to adapt to US college athletics due to whole different cultures including language compared to their home countries.

To properly support ISAs in their experiences here in the US, multiple groups become vital including campus/athletic department support staff (Rodriguez, 2014), coaches (Popp et al., 2010), teammates (Falls and Wilson, 2012), and other international students (Forbes-Mewett and Pape, 2019). Rodriguez (2014) states academic counselors and support staff are vital to the transition and retention of ISAs. A highly supportive academic support program for ISAs can lead to higher satisfaction with their overall academic experience (Trendafilova et al., 2010). Next, coaches are seen as a positive support system, as they usually act as the first point of contact for ISAs prior to their arrival and are typically one of the key reasons a student-athlete attends a certain university (Popp et al., 2010; Hong, 2018). For coaches to provide a positive support system for ISAs, they need to ensure transparency of their programs’ expectations and culture as a coach’s communication and personality are both influential to the value proposition weighed by ISAs when selecting an American institution (Otto et al., 2020). Thus, coaches represent an important individual for ISAs who rely heavily on coaches for help with both on-and-off the court/field issues (Hong, 2018).

#### 1.1.2. International student-athlete adjustment model

The conceptual theoretical model identified to guide this study was the model of adjustment first proposed by Ridinger and Pastore (2000a) and later expanded upon by Popp et al. (2010). The adjustment model was developed to comprehend the experiences of contemporary ISAs’ transition into NCAA athletics. Ridinger and Pastore (2000a) first proposed the adjustment model to understand the transitions of ISAs attending US colleges. A decade after the initial introduction of Ridinger and Pastore’s (2000a) model for adjustment, a significant review of the model was conducted by Popp et al. (2010) due to the shifting landscape of college athletics and the significant increase in the number of ISAs over the time period (100% jump in population). The Popp et al. (2010) findings revealed similarities and confirmation for the adjustment model, however, they updated antecedent measures that demonstrated the modern experiences of ISAs.

The theoretical model was comprised of three different factors: (a) adjustment, (b) antecedent dimensions of adjustment, and (c) outcomes. First, the adjustment factor included five main areas – academic, athletic, social, personal-emotional, and institutional adjustment. Each of these five main areas were seen as driving forces in the ISA’s transition experience (Ridinger and Pastore, 2000a). The adjustment model then moved into the four antecedent dimensions which directly impacted the five areas of the adjustment highlighted above, these included – personal, interpersonal, perceptual, and cultural distance.

The first antecedent was personal, which holds four distinct categories – self-efficacy (Ridinger and Pastore, 2000a), technical competencies (Ridinger and Pastore, 2000a), travel experience (Popp et al., 2010), and sense of adventure (Popp et al., 2010). In the first iteration of the adjustment model, Ridinger and Pastore (2000a) included only two categories – (a) self-efficacy–one’s own perceived ability to perform academically and athletically and (b) technical competency–one’s ability to speak/write English as a non-native English speaker. Building upon this first model, Popp et al. (2010) revised the personal antecedent to include two new factors – previous international travel experience and sense of adventure. The additional factors covered the travel aspect associated with being an ISA. For example, ISAs expressed that their past international travel experience (often associated with sport participation) helped mitigate negative transitions as they were used to exploring new cultures. They also outlined that the transition to college sport was highly associated with a ‘sense of adventure,’ as they were now taking on a new education system and sporting experience.

The next antecedent in the adjustment model, interpersonal factors, refers to relationships with key stakeholders such as teammates, coaches, administrators, and faculty/staff (Ridinger and Pastore, 2000a). All of these stakeholders are important to the adjustment process for ISAs, as creating interpersonal relationships upon arrival to campus can drastically improve the transition experience (Forbes-Mewett and Pape, 2019). Upon the re-examination of the adjustment model by Popp et al. (2010), the researchers’ updated the model and suggested that the importance of the relationship with faculty and staff was minimal. The authors suggested the reason for this finding might be linked to a few issues, including the lack of decision-making in degree, varying learning styles, absences from class due to athletics, and desire to move back home upon graduation. Therefore one of the purposes of this study is to re-investigative the importance of the faculty and staff relationship in the ISAs transition experience.

Perceptual dimensions represent the next antecedent dimension in the adjustment model (Ridinger and Pastore, 2000a). This includes three factors – realistic expectations (Ridinger and Pastore, 2000a), social support (Popp et al., 2010), and family influence (Popp et al., 2010). The first factor, realistic expectation, includes expectations from ISAs on various social supports from their institution such as advising and counseling, as well as their expectations of the university in general, the academic environment, and the athletic program. The two latter factors (social support and family influence) were additions to the model from Popp et al. (2010). For example, the social support aspect includes the ability to rely on an individual’s network for support during the transition period (family, teammates, coaches, etc.). This differs from the interpersonal aspects based on the difference between inter-personal relationships and support systems. While they hold overlapping application toward interactions with key individuals on-and-off campus, they hold varying experiences for ISAs (Popp et al., 2010). The last factor, family influence encompasses the ISA’s school decision-making process and their subsequent homesickness during the transition process.

**Figure 1:**
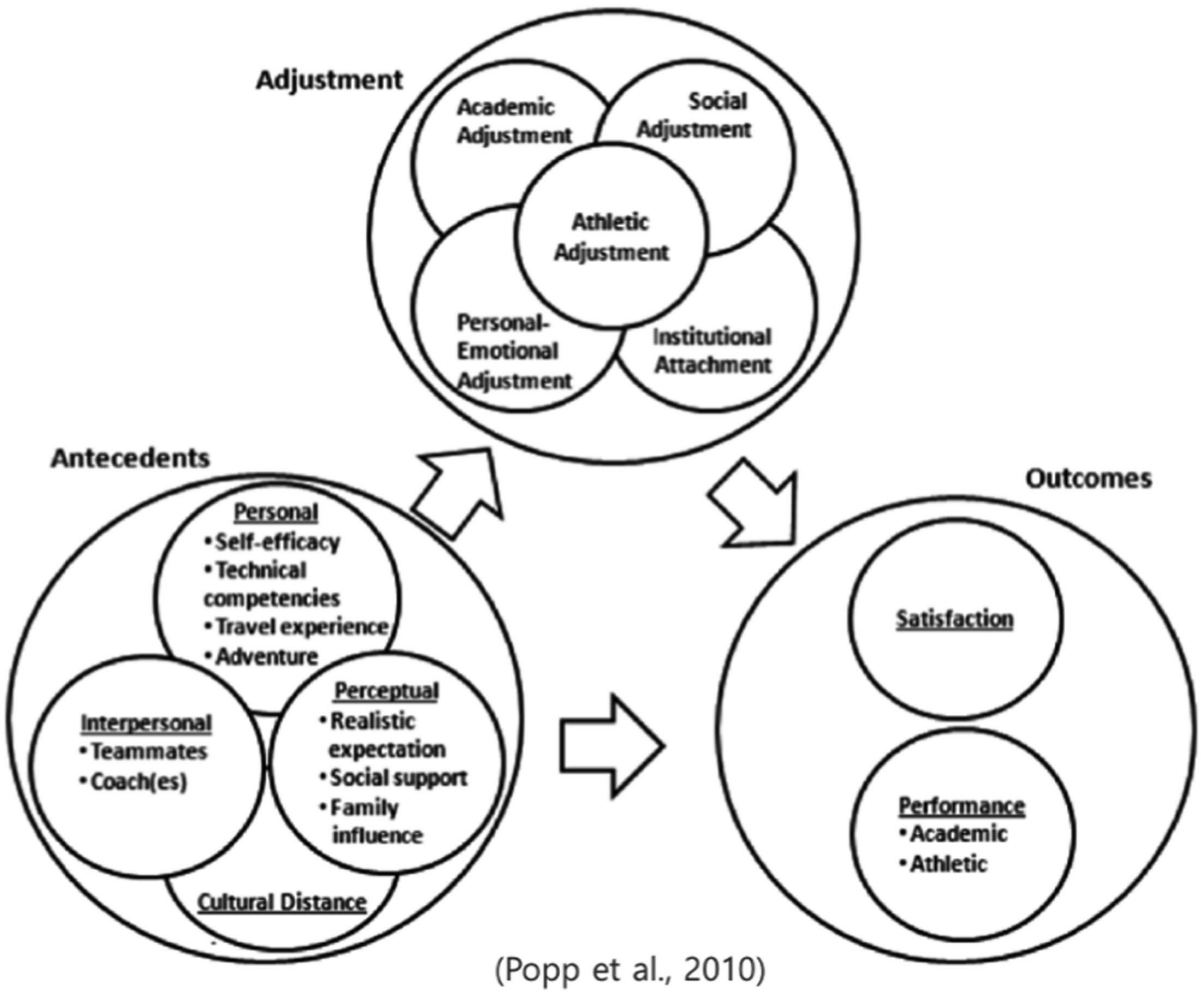
International student-athlete transition adjustment model (adapted with permission from Popp et al., 2010).

The last antecedent, cultural distance, highlights the differences between an athlete’s home culture and the culture they experience on their new college campus (Ridinger and Pastore, 2000a). In the cultural distance antecedent, researchers found the importance of culture surrounding both athletics and academics, as they both played a major role in the transition process. Figure 1 illustrates Popp et al.’s (2010) ISA transition adjustment model.

While Popp et al. (2010) validated the adjustment model for ISAs attending US schools, many factors have changed since this study (e.g., greater increase in the ISA population, large number of ISA transfers, visa policies, conference re-alignment, growth of social media, NIL), demonstrating the importance of re-examining and potentially expanding the ISA transition adjustment model. The purpose of this study was to examine whether the antecedent factors listed in the adjustment model (Ridinger and Pastore, 2000a; Popp et al., 2010) still represent the best indicators of successful transition into college for ISAs. With the growing body of literature surrounding the ISA experience, a re-examination is warranted to help assist in providing a more comprehensive model. This resulted in the following research question: What antecedent factors in the adjustment model are the best indicators for successful transition of international student-athletes into US colleges?

## 2. Materials and methods

### 2.1. Study design

This study utilized a qualitative research design to examine and understand the transitions of ISAs into US colleges. According to Glesne (2016, p. 39), a qualitative study design is useful for “documenting how structures shape individual experiences, and also how individuals create, change, or penetrate the structure that exists.” This approach allows participants to remain in their everyday lives and leads to understanding their real lived experiences (Marshall and Rossman, 2014; Creswell and Poth 2016; Itoh et al., 2017). The qualitative approach utilized in-depth, semi-structured interviews. The semi-structured interview protocol was selected based on its flexibility as it gives researchers the ability to ask probing, in-depth questions with participants when unique themes emerge (Yin, 2009). A qualitative approach and a semi-structured interview protocol allowed for the thoughts, perspectives, and feelings of the participants to be uncovered, which allowed the researchers to enter into and experience the world of the participants (Yin, 1993).

### 2.2. Participants and data collection

The sample was selected based on their status as current or former international women’s tennis players who are either currently attending or previously attended NCAA Division I universities. Tennis is the NCAA sport which represents the highest percentage of female ISAs, as 66% of Division I women’s tennis players are ISAs (National Collegiate Athletic Association, 2022). This is the highest percentage of ISA participation among any sport (male or female), thus the reasoning behind interviewing this specific group of ISAs. Also, according to Parrish et al. (2020), the integration of ISAs in women’s tennis is vital to the success of the sport at the NCAA Division I level. Upon the approval from the Institutional Review Board (IRB), the research team started to create a list of potential participants. To attain the desired sample size, convenience and snowball sampling techniques were used (Roderick, 2006; Bruner et al., 2008). Based on the tenants of convenience sampling, the researchers reached out to current/former women tennis players within their respective networks to help with the recruitment of participants. From here, the snowball sampling technique was used to help identify other potential participants.

Participant recruitment was conducted *via* contacting potential subjects through email, text, Facebook, and Instagram. Upon agreeing to participate in the study, the researchers sent each participant a consent form to review prior to the interview. The researchers then asked participants for their verbal confirmation prior to the start of the interview. All interviews were conducted *via* video conference platforms, with only sound being recorded from these online interviews. To create a sense of familiarity with participants, both Zoom and Microsoft Teams were utilized in the interview process. Interviews were conducted from July 2020 through August 2020 and generally lasted 30 to 60 min. The interview guide used for this study was modified from the past works of Ridinger and Pastore and Popp et al., to best address the antecedent factors impacting ISAs in their transition (see Appendix).

In total, six former and four current Division I female tennis players (*n* = 10) agreed to participate in this study. The participants represented or formerly represented six separate Division I schools and hailed from seven separate countries – Bosnia, Canada, Czech Republic, El Salvador, Russia, Venezuela, and Vietnam. The four current players were all either in their Junior or Senior years, with the six former ISAs ranging from 1 to 5 years post-college. Of the participants, seven attended Power-5 institutions.

### 2.3. Data analysis

All interviews were audio-recorded and transcribed verbatim using the Otter voice recording software. In order to ensure confidentiality throughout the analysis, the researchers assigned pseudonyms to each participant. Braun and Clarke’s (2012) thematic analysis was employed to analyze the interview data. Thematic analysis is a method for systematically organizing, identifying, and offering patterns of meanings/themes across a dataset (Braun and Clarke, 2012). First, the researchers familiarized themselves with the data through reading and re-reading the raw data. Next, the research team employed descriptive coding for the initial codes. During this process each researcher coded two full transcriptions and the results were compared for consistency among the codes. Upon this comparison, a code book was developed based on the initial codes identified. In creating the code book, frequent reference checks back to the work of Ridinger and Pastore (2000a) and Popp et al. (2010) were used as a model to guide the research. As the goal was to connect the actual lived experiences of the participants to the already existing model, this process was seen as most in alignment with past works (Popp et al., 2010). Upon the completion of the first round of coding, the researchers utilized an axial coding technique to further develop the initial codes in categories aligned with the key antecedents of adjustment model. From here, researchers were able to make comparisons to the adjustment model.

## 3. Results

This research study was guided by one research question, which stated “What antecedent factors in the adjustment model are the best indicators for successful transition of international student-athletes into US colleges?” To help address the identified research question, the results were organized according to the four guiding antecedents of the adjustment model. The results are organized by (1) personal, (2) inter-personal, (3) perceptual, and (4) cultural distance. Among these four antecedents the relevant factors were explored based on the findings from interviews. Table 1 outlines the changes in factors over time from the initial iteration of the transition adjustment model to the findings from this study regarding international student-athlete transitions.

**Table 1:** Comparison of International student-athletes transition adjustment models.

Antecedents	Factors		
	Ridinger and Pastore (2000a)	Popp et al. (2010)	Current study
Personal	Self-efficacy Technical competencies	Self-efficacy Technical competencies *Travel experience* *Adventure*	Self-efficacy Technical competencies
Inter-personal	Teammates Coach(es) Faculty/staff	Teammates Coach(es)	Teammates Coach(es) *Faculty* *Students (International, SA’s, Non-SA’s)*
Perceptual	Realistic expectation Social support	Realistic expectation Social support *Family influence*	Realistic expectation Social support
Cultural distance	N/A	N/A	*Nutrition*

### 3.1. International student-athlete transition adjustment model

The study research question addressed the antecedent factors from the adjustment model - personal, interpersonal, perceptual, and cultural distance.

#### 3.1.1. Personal

Under the revised model for adjustment (Popp et al., 2010), four factors represented the personal dimension – self-efficacy, technical competencies, travel experience, and adventure. The first two factors, self-efficacy (perceived ability to perform academically and athletically) and technical competencies (ability to speak/write English as a non-native English speaker) were confirmed, with their three key subcategories - athletic aptitude, academic aptitude, and English proficiency – all underscoring the importance of self-efficacy and technical competencies (Ridinger and Pastore, 2000a). Relative to the more recent additions to the adjustment model from Popp et al. (2010), the travel experience and adventure factors were not confirmed. Participants discussed travel experience in a positive manner, as similar to Popp et al.’s (2010) findings, they previously played in high-level international competitions. However, the participants in this study did not discuss this impacting their decision to attend school in the US or help in their transition experience. Further, the subcategory sense of adventure was also not a factor, as participants expressed excitement in their coming to the US for college and the new beginnings this brought but did not describe it as being a sense of “adventure.” The sentiments from participants were rather business like. They were ready to compete or get things started, rather than being adventurous. Therefore, travel experience and adventure were removed from the adjustment model. This will be further explored in the discussion.

##### 3.1.1.1. Athletic aptitude

The participants in this study discussed how their athletic ability improved upon arrival at their US college. One of the main aspects of this was their technical competencies in comparison to their other teammates. As Jessica (Russia) stated, “I think my confidence level started increasing from the first day of our practice….when we practiced as the entire team….and I saw that I’m, well, if not a lot worse than the rest of the team.” Since the US collegiate tennis structure is drastically different than most amateur and pro leagues, this was a big change for participants. For example, all participants discussed the shift from an individualistic approach to tennis to a greater emphasis on team. As Grace (Vietnam) stated,

“And I was really just surprised. Like how everything worked because I was really individualistic while playing on the pro tour and now it is became more of a team environment.”

While the support and team-oriented practice and match play were seen as positive, the transition into this space was also a challenge to athletic confidence (self-efficacy) as Carson (El Salvador) highlighted, “Practices were very focused on me personally [prior to college], and you know, at the college level practices are team…so that was a huge change.” This quote demonstrated the team focused approach may take some getting used to and suggests ISAs might benefit from coaches adopting similar coaching styles to their home country, at least in the first few months of training.

##### 3.1.1.2. Academic aptitude

The participants also discussed varying levels of academic readiness in their college transition. For example, some participants expressed strong past academic backgrounds, including some education in English as a second language, which led to greater confidence and easier academic transitions. One of these individuals was Tiffany (Venezuela) who stated her academic rigor at the high school level allowed for easier transitions,

“I went to a private Catholic school in Venezuela, really very good academics. Basic ones, like the math and English and all those things you have today, I felt my high school was better.”

Academic confidence from past educational experiences was also expressed when participants held little English-speaking experience prior to arrival. Their own personal academic confidence drove their academic transition experiences. Sarah (Bosnia) stated, “I’ve always been like a 4.0 GPA student, so I perceived myself as an excellent student but I obviously had concerns about if I’m going to be able to learn English. That was the only concern I had before coming here.” However, other participants struggled with the academic transition, as their past schooling opportunities were not equal to those of others. As Claire (Venezuela) mentioned, “I think the level of academic challenge was something that it was hard to adjust to. I think my college, my, high school is kind of easy, I do not know. and then going to college was hard.” This sentiment also indicated the potential differences in higher education systems between the US and other countries.

##### 3.1.1.3. English proficiency

Participants also expressed academic struggles primarily tied to language barriers and English proficiency levels. The classroom was one of the more challenging spaces for the ISAs in this study based on their lack of English proficiency and English being a secondary language. Carson (El Salvador) explained these struggles during in-season competition when the team was missing classes, “Yeah, definitely my first semester was difficult because of that you know not only I was having trouble understanding my classes because of the language but also not being able to be in my classes at all.” Participants also noted the importance of using translating software to assist in their classroom material. Grace (Vietnam) highlighted the struggles of one of her teammates during the transition process, “The Japanese girl she, I remember the first semester. She said she had to sit there and just translate the whole book, so that she understood what it is about.” Similarly, Kathy (Czech Republic) stated, “So, every time I went somewhere I had the translator in my pocket” demonstrating a potential extra layer academically in the transition process for ISAs. The language barrier also impacted relationship building during the initial transition phase for the participants. Kathy (Czech Republic) explained, “Hey, sorry I’m not from here you know, like, I might say [something] wrong but you know what I mean?… I try to apologize for making mistakes.” Claire (Venezuela) mentioned the language barrier leading to the potential feeling of being left out, “A lot of the times I felt that you know language barrier, like I would not understand the joke or whatnot and [it would] be awkward.” The language barrier was potentially also a key component in inter-personal relationships in the transition process, which is further explored in the next section.

#### 3.1.2. Inter-personal

For the inter-personal antecedent, Popp et al. (2010) included only two factors, teammates and coach(es), as they removed the early findings of faculty/staff from their updated transition adjustment model (Ridinger and Pastore, 2000a). While the present study found both teammates and coach(es) to be important factors of the inter-personal antecedent, other key inter-personal relationships assisted in the transition process. Two codes were identified to be important among the inter-personal antecedent, including both faculty and other students (both student-athletes and non-student-athletes).

##### 3.1.2.1. Teammates

All of the participants mentioned experiences with teammates. These relationships were all seen to be positive and were some of the first local interactions upon arrival to campus. The commonality among ISAs and their teammates regarding tennis helped foster these relationships, as their new teammates were willing to do whatever they could to help in the transition process. As Sarah (Bosnia) explained, “I did not know English very well. I remembered that they really tried to help me, even they did not know how to communicate themselves how to help me, because I do not understand them very well.” Amanda (Canada) described the relationship with teammates as family-like, “leaning on my teammates more for I guess like learning to create my own family where I was. So, yeah, leaning on my friends and teammates.”

The participants also discussed the importance of having other international student-athletes as teammates, even when they were not from the same country. As Carson (El Salvador) noted,

four of us were from Latin America so almost like half of the team. So, I think, that was really like a really good thing for me, because of the same kind of like language problem. But I think I had a good relationship with my teammate in general even though I was closer with Latin American players.

This could suggest, recruiting in similar regions can help assist during the transition period. This similar discourse was echoed by Veronica (Venezuela) who discussed how connecting with a fellow Spanish speaking recruit on her visit solidified her decision to enroll, and created an instant connection upon her arrival on campus,

I was really lucky because when I visited my school that I went to I visited with a Mexican, so that was Spanish, we can speak Spanish, and then we both agreed to come to the same school so I knew coming in I had a friend, already.

While most participants discussed the importance of having other ISAs on their team, especially those with similar home country culture and who spoke the same native language, others mentioned this can also create tension between teammates. For example, some participants (*n* = 4) discussed that ISAs with similar first languages and cultures may start to create cliques on teams, leading to segregation of the team and a lack of togetherness. As Claire (Venezuela) stated, “On my team there were some Russian girls [from the same country] that would talk [their native language] among them and I thought that was pretty rude because you could tell that they were talking about other girls on the team or about me.” Overall, these sentiments demonstrated the importance of teammates in the transition experiences of the participants, while also highlighting the unique intricacies of the ISAs experiences regarding relationships with teammates.

##### 3.1.2.2. Coaches

The inter-personal relationship with coaches was found to hold relevance with the participants in this study (*n* = 7). Since coaches were the first contact point for most of the sample, both head and assistant coaches were seen as important support systems. As Kathy (Czech Republic) expressed,

I could have taken the phone and call her. So, she was for me. I felt like they were my like, second parent. You know, because I had a trust in them, and I knew that I can count on them. So, it was amazing.

Since participants were far from home, the sense of security coaches gave athletes was important in the transition process. This was expressed by Tiffany (Venezuela) as her coach helped with a significant portion of challenges associated with recruiting and applying to her institution. For example, “He facilitated everything, everything that I needed to get through such as SAT and the transcripts.”

The participants also emphasized the importance of coaches in their athletic experience. When coaches created connections with players, their transition experience was positive. As Sarah (Bosnia) noted, “He understand me better as a person and my personal goals that I want to say I actually want to play professional tennis. We are both from the first day on the same page.” As such, coaches wear multiple hats during the transition experiences, as they are the central hub for athletics, but also academics and campus information. Further, participants expressed the unique ability of their coaches to blend inter-personal relationships with tennis (a potential commonality between the players and coaches), as Carson (El Salvador) posited,

Once I was there, you know, coach was very nice. I was happy with practices and with my relationship with the coach as well. I feel like you know he was trying me to help me improve my game. So, it was a good relationship overall.

While coaches were found to be an important factor to the experience in the transition process for participants, another key member of the campus experience, faculty, was highlighted by participants.

##### 3.1.2.3. Faculty

The re-emergence of faculty in this model was not expected as Popp et al. did not find sufficient evidence in their 2010 study to include it in the model. This finding in the present study suggests including faculty in the adjustment model is justified. For example, the participants (*n* = 6) discussed their experiences with faculty to be important during their college transition experience. For example, Jessica, (Russia) stated,

I think the main group of people that helped me adjust here were professors. Because I started first of all with their office hours/They’re extremely helpful during the office hours because you can talk not only about the subject, but also they helped me out with the what classes, what major I was going to take, and then what classes I needed to take for that. So, they were open to help in any questions.

Sarah (Bosnia) noted similar experiences,

I have good relationship with faculty members. I really respect my professors. I look to them as more as people who are there to guide you and to help you through the learning process. So, I’m not like afraid to ask for help or to talk about class after the class or during the office hours.

The participants’ responses suggest faculty may be a key component in the academic transition experiences of ISAs. While athletic academic support was noted as important, it did not emerge as a specific theme in this study. The participants in this study did overwhelmingly expressed the importance of connecting with faculty to assist in their overall academic experience, Carson (El Salvador) further expressed this by stating, “You know after class I’ll sometimes go ask a question to my professors and they’ll be very helpful, or in their office hours as well so I had a good experience with them.” Including faculty in this model may help explain the holistic academic transition experience, as the inter-personal relationships between participants and faculty suggest a smoother transition for ISAs in their academic endeavors, which also might take the stress off coaches in this process.

##### 3.1.2.4. Students

Not included in previous transition adjustment models, the results from this study suggest the addition of students under the inter-personal antecedent. While the Popp et al.’s (2010) findings hint at the importance of other international students to help mitigate negative experiences in the transition process, they did not include this in their 2010 model. Among the new factor of students, three important groups emerged – international students, other student-athletes, and non-student-athletes.

First the participants discussed the importance of other international students on campus. While their teammates provided quality support, other international students on campus provided valuable relationships. This was emphasized by Sarah (Bosnia), who said, “international groups…help with some issues which international students can experience” suggesting integrating into the greater international student community may assist in the transition. However, Grace (Vietnam) also discussed the potential setbacks to these relationships, stating “there’s the international group and they see there are others who speak the same language and they usually found to go and spend more time with them, which do not really help their English.” While connections with other international students are important, it may not enhance language competencies or ISAs integration into the great campus culture. Thus, the findings here suggest a balance of relationships for ISAs among international students can help mitigate negative transition experiences but may be detrimental if networking does not expand beyond this group.

Next, the participants in this study (*n* = 6) highlighted their relationships with other student-athletes as important to their transition experiences. There was evidence to suggest that the similarities surrounding sport experiences created commonalities between ISAs and other student-athletes (both international and domestic students). Veronica (Venezuela) explains this further,

they were amazing to me. I feel that we are the same family even though we are not from the same sport. We are a student athlete. So that’s something very unique that we had on my school. We were supporting each other so 100%. I had a great experience with that.

Since student-athletes from other teams have similar lived sport experiences, they can almost act as further-removed teammates. As all participants in this study participated in tennis, with relatively smaller team sizes, other student-athletes might prove important for ISAs. One way to integrate student-athletes from separate teams together is by utilizing shared spaces, which can include residence halls, dining halls, academic centers, and weight rooms. For example, Tiffany (Venezuela) mentioned,

There were a couple of dining halls that you can just eat at if you’re a student athlete. You cannot get in if you’re just a student. So, it was very easy to hang out with others student athletes in the same environment, pretty much, after practice or like, even at night.

Therefore, these findings highlight the need for ISAs to immerse themselves in the overall athletics campus culture through making inter-personal connections with other student-athletes beyond their own teammates. These extended networks outside their individual sport may help ISAs make a smoother transition.

Lastly, one group highlighted by the participants under the student factor was non-student-athletes. The non-student-athlete population on campus also had the ability to enhance the ISA lived experiences during the transition process. The participants in this study discussed the importance of attending classes with non-student-athletes, which led to important inter-personal connections. As Sarah (Bosnia) stated,

I spend a lot of work time with non-student athletes, because they are more dedicated to school and they have more time to dedicate to school then student athletes. And I would say like, [it was a] very positive experience because they helped me a lot for some of my classes. We had study groups together.

However, some participants highlighted feeling scared or hesitant to engage in friendships with non-student-athletes as this was not seen as the norm on their campus. As Veronica (Venezuela) stated,

Non-student-athletes - they were amazing. I had such a blast with them. Okay. I feel that they see you like a god. Oh my god you’re student-athletes. I feel that, of course, in my freshman year I was more shy in our small clique of athletes. But over the years when I went to junior and senior year I had more non-student athletes as friends. And because I opened myself more, I became friends with people from my classes. And I will say yeah I absolutely changed in my senior year. I had more non-student-athlete friends, absolutely 100%.

This illustrates that ISAs might be reluctant to make connections with non-student-athletes on campus based on perceived differences. When they engaged and connected with this large population, however, they had the ability to drastically improve their experiences both personally and academically.

#### 3.1.3. Perceptual

Perceptual antecedents of the adjustment model were also present in this study, similar to past models (Ridinger and Pastore, 2000a; Popp et al., 2010). The perceptual antecedents were first introduced as realistic expectations and social support (Ridinger and Pastore, 2000a) with a later addition of family influence (Popp et al., 2010). The present study confirmed the past factors of realistic expectation and social support. However, the social support perceptual factors only included minimal support for academic counselors, a prominent theme from past studies. Social support, as highlighted above, rather focused on coaches, teammates, faculty, and other students. The family influence factor was also not supported by the majority of participants in the present study. While some did mention family in the decision to attend college in the US, the use of technology bridged the gap in feeling homesick (Kelly et al., 2021), which lessened the identification of family in the transition process. This study’s results suggest the updated revision for this model should exclude family influence in the adjustment model. While the authors are not arguing family does not play a role in the overall experiences of ISAs on US college campus, the suggestion is that the family unit, due to increased access to family through technological advancements, does not play as a substantial role in the transition process.

##### 3.1.3.1. Realistic expectations

The realistic expectations of the participants in this study centered around their lack of knowledge of the structure of higher education in the US. The participants expressed athletic reasons for attending college in the US, with little understanding of the inner workings of their campuses. Some participants (*n =* 4) reflected on their past tennis experience, as this was a potential strong driver in choosing to come to the US to compete in college tennis. For example, the participants discussed how the goals of playing professionally were potentially out of reach. However, they still wanted to compete at a high level while also getting a degree to further their career post-college and post-sport participation. As Tiffany (Venezuela) stated,

First, I didn’t want to go to college. That was not in my plans. I wanted to go professional, so in my senior year that decision was made with my parents and my coach to lay out the options that I had… The professional route was not very viable. It was not very feasible, so my senior year, a bunch of colleges started reaching out to me.

One participant (Grace, Vietnam) even mentioned this as one of her talking points when other potential future ISAs reach out for her advice. As she highlighted in her interview, she actually lost a year of eligibility based on her lack of knowledge of the NCAA system:

In Asia, a lot of them don’t know, including me back then. I just agreed or trusted whatever the coaches were saying, and I just didn’t know what to ask and I see that common theme…. So, I try my best to explain to them and, and if they were telling me about you know their plan of going pro or anything, I would make them aware of eligibility [rules].

This demonstrates that while coaches are potentially doing their best to communicate with ISAs on eligibility and resources, the messages may not always be received or understood. Grace further suggested the need for current and former ISAs, especially in the popular sport of tennis, to act as liaisons for younger players in their home countries. The ISAs could help guide the recruits in making smart decisions for their futures by providing first-hand information on what to realistically expect in their new sport environment.

Some participants (*n* = 5) even discussed having little to no information prior to enrolling. For example, Kathy (Czech Republic) mentioned “Okay, so I did not know what to expect there because I did not know how the system work before I came here.” This comment demonstrates a potential disconnect between coaches, athletes, and the athletic department itself in preparing ISAs for success. In the athletic realm, Sarah (Bosnia) discussed her lack of knowledge on what to expect regarding competitions and events. The coaches in her case potentially oversold the reach and experiences of their program to the future ISA,

They promised at the very beginning that they will provide us additional competition outside of the college. I was expecting to pick up a couple pro tournament, which really didn’t happen. I don’t know if it because of schedule conflicts or is it because of lack of budget or whatever.

Overall, this demonstrates that while ISAs might be receiving information in the virtual setting from schools and coaches, the information might not be meeting the needs of the ISA population. Since the ISA population is so diverse, athletic departments might benefit from providing more specific information to ISAs during the pre-enrollment period. For example, a school might provide resources to ISAs in varying languages to assist in the comfort level of their programs, both athletically and academically.

#### 3.1.4. Cultural distance

The participants (*n* = 7) also confirmed the cultural distance antecedent of the adjustment model. Overall, the sentiment toward differences between participants’ home country culture and US culture effected the transition experience. The participants noted the struggles with social interactions during their transition, as Kathy states,

I’m not really sure if are the true friendships you build there. I don’t have really friends from US just and I don’t think like I did something wrong or they did something wrong. But like the talks they were like more formal or you don’t go deep into the topic.

While more minimal, the interaction differences were also discussed by participants (*n* = 4). For example, during her transition, Jessica indicated simple interactions such as “what’s up” were taken as negative interactions (thought she was in trouble) based on her home culture, whereas the interactions are simple passing conversations in US college culture. However, as time elapsed in the transition process, Carson (El Salvador) noted that she became more comfortable with these types of interactions. As such, the more interactions individuals can experience, the quicker they will be able to start becoming more comfortable in their new culture.

The participants also emphasized the importance of food and nutrition in their transition process. This aligns with past findings (Popp et al., 2010), therefore, the suggestion to add nutrition to the factors under cultural distance aspect is warranted. The majority of participants (*n* = 7) discussed the importance of understanding the differences between ISA home food culture and the US food culture. For example, participants regularly discussed struggles with weight gain and consumption patterns. As Tiffany (Venezuela) stated, “Here food is terrible. I gained some weight. When I got here, because how they prepared the food or where the food is coming from, even if it was in the same thing as I was eating in Venezuela.” The participants also struggled with portion control and finding healthy options on campuses as most of the time their scholarship included on-campus meal plans, pushing them to frequently eat on-campus. This was further discussed by Sarah (Bosnia),

Food affected my adjustment here because like freshman year I couldn’t adjust. I couldn’t find healthy options and portions are way bigger than in my country. So, it took me whole two years to lose that weight, and to come back again on a good weigh-in. I mean I was eating healthy but I was eating bigger portions.

On most college campuses, fast food type options are more readily available for ease of access then healthy options, which can lead to challenges with food and nutrition. As Veronica (Venezuela) stated, her teammates sometimes struggled with finding healthy options on campus, “not speaking by myself, like, I know my team struggled with eating well. Not like we were eating fast food, none of that, but we were not eating the right way.” The participants also highlighted their lack of knowledge on food, which suggested the nutrition factor should be added to the ISA transition experience.

## 4. Discussion

This study’s purpose was to re-evaluate the international student-athlete adjustment model for the 21st century ISA. Overall, this study confirmed the four established core antecedents from the ISA adjustment model and suggested changes within the underlying factors in the inter-personal, perceptual, and cultural distance antecedents. The transition experience for ISAs is multi-faceted, leading to the importance of re-investigating the experiences of ISAs over time (Popp et al., 2010; Hong, 2018; Otto et al., 2020).

While this study confirmed the key antecedents from the framework of the original (Ridinger and Pastore, 2000a) and revised adjustment models (Popp et al., 2010), it also suggests variations should be made to better encompass the holistic experience of the ISAs in their transition periods. For example, under the personal antecedent, two factors – past travel experience and sense of adventure – should be removed (Popp et al., 2010). While the results from Popp et al. (2010) suggested the importance of travel and adventure, this was not the case in the findings of this study. The participants in this study all highlighted international travel during their youth sport participation (heightened travel opportunities due to larger numbers of national tennis tournament, in comparison to other sports), however, this travel experience was not indicated as a prominent variable in the transition process for the ISAs in this study. They also did not express any sense of adventure. Most arrived on campus a few days before classes started, thus, they did not have time to ‘explore’ or feel ‘adventure’ as they had to get right to work, as one participant mentioned in a ‘business like’ manner. Therefore, the authors do not disagree that travel might be important to ISAs’ sporting experiences prior to attending the US for college, but it does not impact the transition experience, based on the findings in this study.

This study also found key additions to the inter-personal antecedent of the adjustment model. The participants all discussed the importance of interacting with individuals on campus other than just their coaches and teammates. While coaches and teammates were found to have central impact on the transition experience, this study suggests two further additions to the model. First, the addition of students (international students, student-athletes, and non-student-athletes) is warranted. This aligns with past findings, as students outside of the ISA’s team have been found to enhance the overall experience of ISAs in US colleges (Foo et al., 2015; Forbes-Mewett and Pape, 2019; Otto et al., 2020). The findings from this study suggest students may also hold importance in the transition process. However, one potential drawback occurs when other international students are part of inter-personal relationships, as ISAs might struggle to integrate into the campus culture. As Montgomery (2010) highlights, relationships between international students may temporarily foster positive experiences in the transition period, but they may lead to a lack of integration to the campus culture. Thus, the populations of domestic student-athletes and non-student-athletes are also vital to the experience for ISAs (Otto et al., 2020). Overall, this study suggests some of the most salient types of inter-personal relationships on campus exist with other students, both international and domestic.

This study also shed light on another key finding - the suggestion that faculty members at institutions play a major role in the ISA transition. The finding of faculty being influential in this process was also found by Ridinger and Pastore (2000a), but not confirmed in later studies (Popp et al., 2010; Hong, 2018; Otto et al., 2020). For example, while Otto et al. (2020) discussed the challenges of understanding the educational system for ISAs, their participants did not indicate faculty as an important variable in the transition process. As Newell (2015) argues, ISAs often come to the US for the mix of academics and athletics which are not available in their home country. This leads to potentially stronger academic identity than other student-athletes, which may further explain the stronger ties with faculty found in this study. Furthermore, faculty have the ability to mitigate potential stressors for the ISAs, for example, missing class for athletic travel and having questions about assignments. These findings contradict that of Lee and Opio (2011), who found ISAs from African countries sometimes felt targeted by faculty. This highlights some inconsistencies when researching the ISA population, as individuals may hold unique variability in their experiences based on ISAs home country of origin, race, religion, and sport participation. To best address the needs of ISAs, similar to coaches and support staff, faculty need to be cognizant of the needs of ISAs to best encompass a positive and supportive academic environment.

The participants in this study also suggested athletic academic support was a great resource during their transition, however, those support systems were not as meaningful as the inter-personal relationships with faculty members. For example, higher achieving academic ISAs noted that they held less meaningful and impactful interaction with athletic department academic support and that faculty offered key resources to further success in the classroom. This could be a shift from the findings from Popp et al. (2010) who suggested that ISAs might be distant from faculty due to not being able to select their major/degree path. Thus, this might hint that ISAs have increased their decision-making power for majors, putting less pressure on ISAs to major in a subject outside their desired field, such lessening the need to utilize academic advisors.

The current transition adjustment model should also include the factor of nutrition under antecedent of cultural distance. Similar to the preliminary findings from Popp et al. (2010) and more recently Otto et al. (2020), this study found unique cultural differences regarding food, including portion control and selecting healthy options. This is not a unique experience for ISAs in the college experience, as domestic and international students both grapple with handling their own food choices (Chaiet, 2018). However, the ISA population is being asked to perform at high levels athletically and are usually restricted to on-campus meal options (see Dhillon et al., 2019 for an overview of the unhealthy options colleges offer in on-campus eateries). Limited access to healthy food choices and education surrounding nutrition potentially leads to additional challenges to overcome in the transition process.

As Shriver et al. (2013) suggests, student-athletes often have trouble with their eating habits on campuses. Introducing information on nutrition and food access may therefore be especially important in the adjustment model. To date, many colleges athletic departments have potentially started to address this issue, as we have seen a dramatic shift with athletic departments hiring more nutritionists/dietitians to their staffs (Collegiate and Professional Sports Dieticians Association, 2015; Riviere et al., 2021). As the research regarding the importance of nutrition and athletic performance expands, the need to assist ISAs in their nutrition transition experience is warranted. Especially considering ISAs face unique challenges with food and nutrient based on cultural differences in food consumption and prep. Therefore, nutrition should be added to the cultural distance antecedent, and should be a major focus in the transition process to help mitigate negative experiences for ISAs.

### 4.1. Future research/implications/recommendations

This study has several implications, for both research and practice. For example, the participants in this study competed specifically in women’s tennis, leading to the findings to potentially be limited to this population. Examining different sports would add to the literature in this area. A second potential research area to explore is to continue to build on the experiences of the ISA post-college transition. Future research should delineate the differences in domestic and international student-athletes’ post-college transitions. This research could potentially lead to more diverse programming in the post-college transition process, with focuses on internships, career opportunities, and visa eligibility.

In practice, this study suggests a few key implications to enhance the ISA experience in their transition process. The first would be to introduce specific education for ISAs in both language proficiency and health and nutrition. These two factors were found to be important in the transition experiences of ISAs; therefore, introducing educational programming in these areas can help potentially mitigate negative experiences. For example, ISAs without English classroom training should be given the option to enroll in free NCAA-sanctioned English proficiency courses up to 6 months prior to arriving on-campus. The ISAs in this study did not describe having an option like this, yet all discussed struggles their English language proficiency upon their arrival. For health and nutrition, either the NCAA or athletic departments themselves should introduce ISAs to healthy eating and nutrition trainings. These trainings will focus on understanding the differences in meal preparation in the US food system and allow the ISAs the ability to make informed decisions on their eating patterns.

Another important finding in this study, which furthers the theoretical framework of the ISA adjustment model focused on the, academic experience. Adding the faculty component back to the model furthers the conversation that the ISA experience is an ever-evolving experience and one study (or a group of multiple studies) might never fully encompass the holistic experiences, as ISAs hold quite diverse backgrounds. However, this study suggests that potentially the best resource for ISAs academically, lies with faculty support. This challenges the idea that athletic departments must do everything for their student-athletes in academic programming, especially for ISAs, who potentially hold vastly different educational experiences, in comparison to their domestic student-athlete counterparts. While academic programs housed in athletics are great for ISA academic development, it is no secret that the primary focus of athletic academic support is often to make sure student-athletes are eligible and on track for graduation. While not all students are the same, and some ISAs might need the internal support from academic counselors (especially concerning eligibility), ISAs should be encouraged to seek more positive relationships with professors and classroom TAs, potentially improving their transition experience.

## 5. Conclusion

Overall, this study focused on better understanding the ISA experience through the International Student-Athlete Adjustment Model. This study illuminated the unique experiences of the 21st century ISA and suggests revisions to the ISA transition model may need to be addressed. Specifically, this study recommends ISAs should engage more with the wider campus community include creating inter-personal relationships with other students and faculty, which may improve their holistic transition experience. This study also suggests two major barriers in the transition process of ISAs, nutrition and language, should become central focuses in athletic departments ISA transition programming.

## Data availability statement

The datasets presented in this article are not readily available due to the privacy of research participants. Requests to access the datasets should be directed to nicholas.swim@louisville.edu or youngjik.lee@kookmin.ac.kr

## Ethics statement

The studies involving human participants were reviewed and approved by University of Louisville. The patients/participants provided their written informed consent to participate in this study.

## Author contributions

All authors were involved in designing the study and procedures. NS and YL conceptualized the study and analyzed the data. MH reviewed and edited the manuscript. All authors have read and agreed to the published version of the manuscript.

## Conflict of interest

The authors declare that the research was conducted in the absence of any commercial or financial relationships that could be construed as a potential conflict of interest.

## Publisher’s note

All claims expressed in this article are solely those of the authors and do not necessarily represent those of their affiliated organizations, or those of the publisher, the editors and the reviewers. Any product that may be evaluated in this article, or claim that may be made by its manufacturer, is not guaranteed or endorsed by the publisher.
