# Supplementary material for: “Sorry, I’m not from here!”: Female international student-athletes’ transitions into college athletics in the United States
Source: Front Psychol. 2023 Mar 13;14:1103194. doi: 10.3389/fpsyg.2023.1103194 (PMC10042176; doi:10.3389/fpsyg.2023.1103194)
Supplement: Supplementary file 1 [file Data_Sheet_1.pdf]

6. Appendix

Interview guide

1. Can you please share how your **personal factors** positively/negatively affected your adjustment to US college as a student-athletes?

Probe:

- **Self-efficacy**
  - What was your confidence levels of athletic performance prior to attending current school?
  - **(Former Student-Athlete Only)**
    - Did your confidence level increase during your time in US college athletics?
  - What did you perceive your academic abilities in high school? (high school GPA)
  - **(Former Student-Athlete Only)**
    - Did your perceived academic abilities improve upon attending a US college?
- **Technical Competencies**
  - What was your competency of English as a non-native English speaker?
  - Discuss any current problems with communication with coaches/teammates/support staff.
  - **(Former Student-Athlete Only)**
    - Did your English competency improve during your time attending a US college?
- **Travel**
  - What was your previous international travel experiences?
  - What was your level of sense of adventure in attending a US College/University?
- **Could you address any other personal factors that affected your adjustment?**

2. Can you please share how your **interpersonal factors** positively/negatively affected your adjustment to US college as a student-athletes? (Relationship building)

Probe:

- **Teammates**
  - How was your interaction with your teammates upon arrival to a US College/University?
  - **(Former Student-Athlete Only)**
    - Did this shift or change over your playing career?
- **Coach(es)**
  - What was your interaction with the coach(es) during the recruiting process?

- How did the relationship build after you arrived in the US?
- What is your relationship like in comparing your Head Coach v. Assistant Coach?
- **(Former Student-Athlete Only)**
  - Did any of these relationships change over your playing career?

➢ **Faculty/staff**

- What is your relationship with academic support?
- What is your relationship with athletic trainers/strength coaches?
- **(Former Student-Athlete Only)**
  - Did any of these relationships change over your playing career?

➢ **Student body**

- What is your interaction/relationships with other student-athletes on campus?
- What is your interaction/relationship with non-student-athletes on campus?
- **(Former Student-Athlete Only)**
  - Did any of these relationships change over your playing career?

➢ **Family**

- How did your family impact your decision to come here to compete?

➢ **Could you address any other interpersonal factors that affected your adjustment?**

3. Can you please share how your **<u>perceptual factors</u>** positively/negatively affected your adjustment to US college as a student-athletes? (What you expected before attending)

Probe:

Current/Former Student-Athletes

➢ What were your expectations of what the University and athletic program will be like?

➢ What were your expectations of what sort of social support, such as counseling and advising? Any programs at your University help with this process?

➢ Were there any other perceptual factors that affected your adjustment?

Former Student-Athletes (ONLY)

➢ Did the University and athletic program meet your perceived expectations?

➢ Did the social support, such as counseling and advising meet your perceived expectations?

➢ Did the University meet your expectations to properly prepare you to graduate into a position in your academic field?

➢ Did any have any other perceived expectations that you feel you would like to discuss?

4. Can you please share how cultural distance factors positively/negatively affected your adjustment to US college as a student-athletes? (Differences in culture)

Probe:

- Was there difference between the home culture and the culture found on the college campus? Were these societal or part of a culture gap?
- Geographical distance (home-sick)
- Was there any other cultural distance factors that affected your adjustment?

5. If you had to list five major things that mostly affected your adjustment, what would they be?

6. Can you please share how the corona virus has affected your college experience? (**Current Student-Athletes only)**

- How did your coach/advisors/support staff communicate with you upon moving online this past spring?
- What has been your experience upon moving online? Do you feel being an international student-athletes has created different challenges then other student-athletes?
- How has your University communicated with you about their plans for the fall semester?
- Upon the announcement from the federal government requiring International students to be in-classrooms, did your school/coaches/support staff communicate with you?

7. What are your expectations/experiences about transition after college?

Current Student-Athletes

- Have your life goals shifted since first attending a US college?
- Do you plan on staying in the US or traveling back to your home country?
- Do you feel prepared in your ability to transition after college?
- Could you address any other concerns/excitements about the transition after college?

Former Student-athletes

- Did you stay in the US or move to a new country?
- How has your transition after college been?
- Do you feel you were ready to make the shift from college athlete to the work-force(physically and or mentally)?
- What has been the hardest challenge in your transition out of college?
- What do you feel YOU could have done to make the process better?
